# Direct-Acting Antiviral Drug Modulates the Mitochondrial Biogenesis in Different Tissues of Young Female Rats

**DOI:** 10.3390/ijms242115844

**Published:** 2023-10-31

**Authors:** Hala A. Hafez, Ali M. Atoom, Rana H. M. Khafaga, Sara A. Shaker, Maher A. Kamel, Nagwa M. Assem, Shimaa A. Mahmoud

**Affiliations:** 1 Department of Biochemistry, Medical Research Institute, Alexandria University, Alexandria 21561, Egypt; ranahassanmahmoud@alexu.edu.eg (R.H.M.K.); sarah.a.shker@alexu.edu.eg (S.A.S.); nagwa.assem@alexu.edu.eg (N.M.A.); shimaa.abdelraheem@alexu.edu.eg (S.A.M.); 2 Department of Medical Laboratory Sciences, Faculty of Allied Medical Sciences, Al-Ahliyya Amman University, Amman 19111, Jordan; a.atoom@ammanu.edu.jo

**Keywords:** DNA polymerase γ, hepatitis C, mitochondrial DNA, Sofosbuvir

## Abstract

(1) Background: Hepatitis C virus (HCV) infection is endemic in Egypt, with the highest prevalence rate worldwide. Sofosbuvir (SOF) is a nucleos(t)ide analog that specifically inhibits HCV replication. This study aimed to explore the possible effects of the therapeutic dose of SOF on the mitochondrial biogenesis and functions of the liver, muscle, and ovarian tissues of young normal female rats. (2) Methods: This study was conducted on 20 female Wistar rats, classified into two groups, the control group and the exposed group; the latter was orally supplemented with 4 mg/kg/day of SOF for 3 months. (3) Results: The exposure to SOF impairs mitochondrial biogenesis via mitochondrial DNA copy number decline and suppressed mitochondrial biogenesis-regulated parameters at mRNA and protein levels. Also, SOF suppresses the DNA polymerase γ (POLG) expression, citrate synthase activity, and mitochondrial NADH dehydrogenase subunit-5 (ND5) content, which impairs mitochondrial functions. SOF increased lipid peroxidation and oxidative DNA damage markers and decreased tissue expression of nuclear factor erythroid 2-related factor 2 (Nfe2l2). (4) Conclusions: The present findings demonstrate the adverse effects of SOF on mitochondrial biogenesis and function in different tissues of young female rats, which mostly appeared in ovarian tissues.

## 1. Introduction

The hepatitis C virus (HCV) is a severe human infection that is spread by blood and is a significant public health concern. The World Health Organization (WHO) estimates that 3–4 million new cases of infection occur each year. Most acute infections progress to cirrhosis and hepatocellular carcinoma (HCC) [1]. Egypt has the highest global burden of HCV infections, with an estimated prevalence of around 4.5% to 6.7% [2].

A new national strategy was proposed by the Egyptian government to control the HCV epidemic in Egypt with a great capital fund supported by the WHO organization, entitled “The Plan of Action for the Prevention, Care and Treatment of Viral Hepatitis 2014–2018”, and it promoted SOF as the primary treatment. SOF is a direct-acting antiviral (DAA) drug that acts as nucleos(t)ide analogs. It directly inhibits the replication of HCV, so it inhibits nucleotide polymerase by interfering with the replication of the virus’s genetic material and preventing the development of new HCV [3].

Mitochondrial toxicity is a well-known drawback among nucleoside analogs. Many DAA drugs used for the treatment of human immunodeficiency virus, hepatitis C or B infections, have been reported to interfere with mitochondrial DNA (mtDNA) replication and transcription, and may affect the activity of DNA polymerase γ (POLG) and mitochondrial topoisomerases [4,5]. POLG seems to be the only known DNA polymerase in animal mitochondria, and it is necessary for the transcription, replication, and maintenance of the mtDNA. Mitochondrial RNA transcription and DNA replication are intertwined; both RNA transcription and DNA replication control mtDNA copy numbers. The POLG replaces the RNA polymerase and starts DNA replication from the RNA primers; perhaps POLG has a higher affinity to the transcription bubble than the RNA polymerase once RNA polymerase dissociates from the template [5]. POLG is susceptible to nucleos(t)ide analogs that treat HIV-1 infections, which contributes to mitochondrial stress and toxicity [6]. Mitochondrial stress is implicated in many disease manifestations through impaired adenosine triphosphate (ATP) production and excessive reactive oxygen species (ROS) generation, which may lead to organ failure, especially in high energy-demand organs [7,8].

The mtDNA is very susceptible to oxidative damage due to the lack of histones and efficient repair mechanisms in mitochondria, besides the proximity of mtDNA to the oxygen radical-producing electron transport chain (ETC). However, SOF is considered safe regarding mitochondrial toxicity [9], Jin et al. (2017) demonstrated that the structurally diverse ribonucleoside analogs favipiravir, balapiravir, SOF, and others may cause mitochondrial toxicity [10].

The mass treatment of Egyptian patients with SOF, especially involving young females of child-bearing age, raises many concerns about the possible long-lasting side effects of SOF on mitochondrial functions and biogenesis, which may affect the metabolic homeostasis in the organs of the females and/or their offspring. So, we aimed to explore the possible side effects of SOF on the mitochondrial biogenesis and function of young female rats’ ovaries, liver, and muscle tissues.

## 2. Results

### 2.1. Glucose Homeostasis Parameters

The results of glucose homeostasis parameters (fasting blood glucose (FBG), insulin and homeostasis model of assessment insulin resistance index (HOMA-IR)) are shown in “Table 1”. The female rats exposed to SOF showed significantly lower FBG levels compared with the normal unexposed rats (*p* < 0.001) by about 25.6%. However, no significant difference was found between the two groups regarding serum insulin and the insulin resistance index (HOMA-IR) (*p* = 0.126, 0.076, respectively).

### 2.2. Serum Lipid Profile

The female rats exposed to SOF had a significant increase in triglyceride (TG) level compared with normal rats (*p* < 0.001) by about 29.6%. The SOF-exposed females have significantly higher total cholesterol (TC) levels by about 100% compared with the control rats (*p* < 0.001). While high-density lipoprotein-cholesterol (HDL-C) showed no significant changes in the SOF-exposed females (*p* = 0.22), the low-density lipoprotein-cholesterol (LDL-C) showed a significant increase by about 545.2% in SOF-exposed females compared with the control female rats (*p* < 0.001) (Table 2).

### 2.3. Serum Liver Enzymes Activity and Kidney Function Tests

The female rats exposed to SOF showed significantly higher serum alanine aminotransferase (ALT) and aspartate aminotransferase (AST) activities by about 56.2% and 32.9%, respectively, compared with normal rats (*p* < 0.001, 0.006, respectively). Kidney function tests showed a significant increase in urea level and a non-significant increase in creatinine level in the SOF-exposed females by about 12.3 and 22.6%, respectively, compared with the control female rats (*p* = 0.021, 0.071, respectively). (Table 3).

### 2.4. Serum Luteinizing Hormone (LH) and Follicle-Stimulating Hormone (FSH)

**Figure 1:**
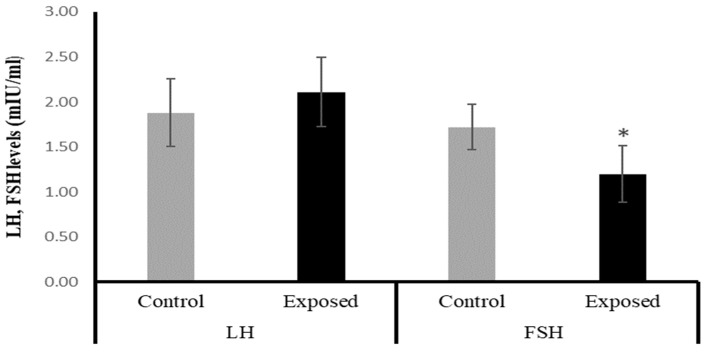
The serum luteinizing hormone (LH) and follicle-stimulating hormone (FSH) levels in control and SOF-exposed female rats. The data were indicated as Mean ± SD. 10 rats in each group. *: Statistically significant difference compared to the control group by Student *t*-test, *p* < 0.05.

The female rats exposed to SOF had significantly lower FSH levels by about −30% compared with the control rats (*p* = 0.001). On the other hand, the LH level showed a mild increase in the SOF-exposed females compared with normal rats by about 12% (*p* = 0.193). (Figure 1).

### 2.5. Tissue Content of Malondialdehyde (MDA)

**Figure 2:**
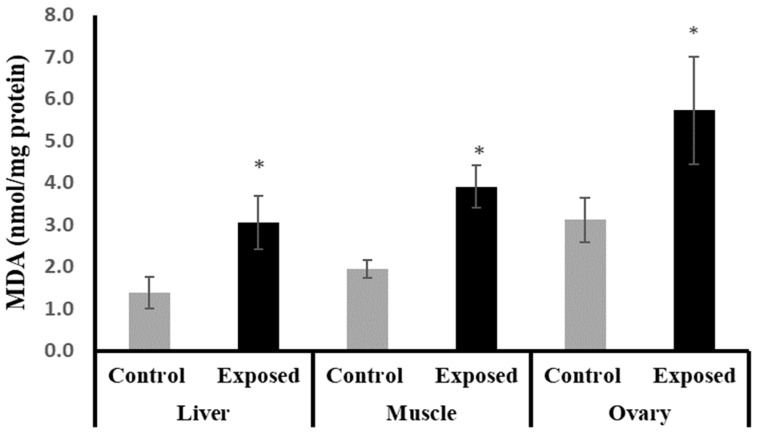
The malondialdehyde (MDA) contents in control and SOF-exposed female rats’ liver, muscle, and ovarian tissues. The data were indicated as Mean ± SD. 10 rats in each group. *: Statistically significant difference compared to the control group by Student *t*-test, *p* < 0.05.

The female rats exposed to SOF showed significantly higher MDA content compared to normal rats in liver, muscle, and ovarian tissues by about 122.25, 100, and 83%, respectively (*p* < 0.001) (Figure 2).

### 2.6. 8-Hydroxy Deoxyguanosine (8-OHdG) in Different Tissues

**Figure 3:**
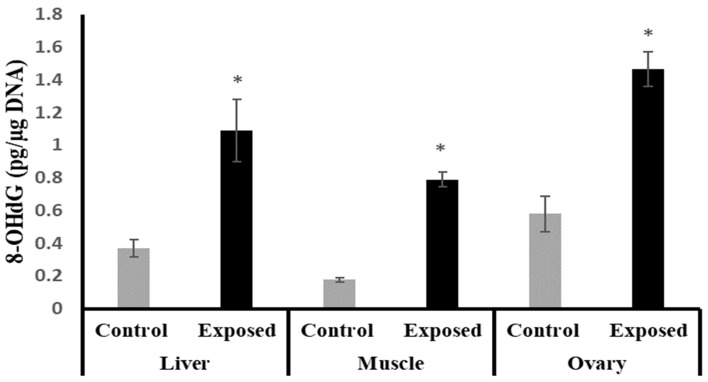
The 8-Hydroxy deoxyguanosine (8-OHdG) levels in control and SOF-exposed female rats’ liver, muscle, and ovarian tissues. The data were indicated as Mean ± SD. 10 rats in each group. *: Statistically significant difference compared to the control group by Student *t*-test, *p* < 0.05.

The liver, muscle, and ovarian tissues of SOF-exposed female rats showed significantly higher 8-OHdG levels compared with normal female rats by about 196.9, 349.7, and 152.9%, respectively (*p* < 0.001) (Figure 3).

### 2.7. Tissue Expression of Nuclear Factor Erythroid 2-Related Factor 2 (Nfe2l2)

**Figure 4:**
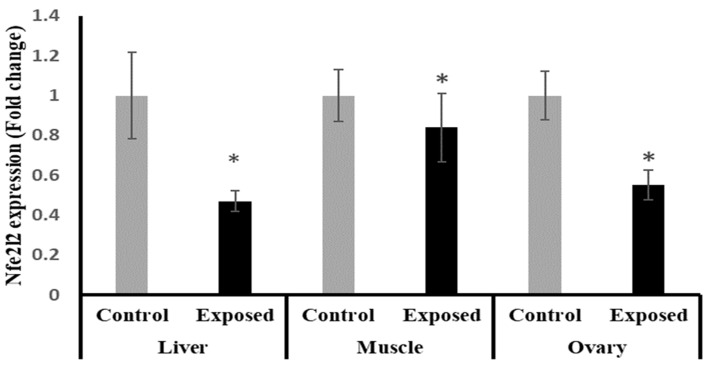
The nuclear factor erythroid 2-related factor 2 (Nfe2l2) expression in control and SOF-exposed female rats’ liver, muscle, and ovarian tissues. The fold change mRNA level vs. control. The data were indicated as Mean ± SD. 10 rats in each group. *: Statistically significant difference compared to the control group by Student *t*-test, *p* < 0.05.

The results of Nfe2l2 expression showed significantly lower expression in liver, muscle, and ovarian tissues in female rats exposed to the therapeutic dose of SOF drug compared to normal rats, and by a percentage of change of −55, −17, and −45% (*p* < 0.001, 0.021, < 0.001), respectively (Figure 4).

### 2.8. Citrate Synthase Activity in Different Tissues

**Figure 5:**
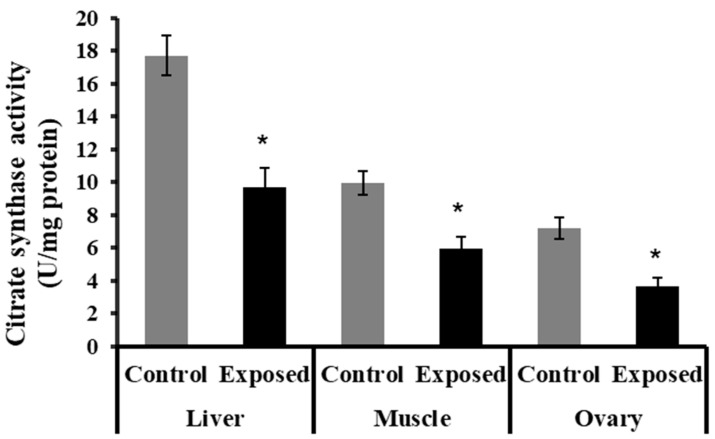
The citrate synthase activity in control and SOF-exposed female rats’ liver, muscle, and ovarian tissues. The data were indicated as Mean ± SD. 10 rats in each group. *: Statistically significant difference compared to the control group by Student *t*-test, *p* < 0.05.

The liver, muscle, and ovarian tissues of SOF-exposed female rats showed significantly lower citrate synthase activity content compared with normal rats (*p* < 0.001) (Figure 5).

### 2.9. NADH Dehydrogenase Subunit-5 (ND5) Contents in Different Tissues

**Figure 6:**
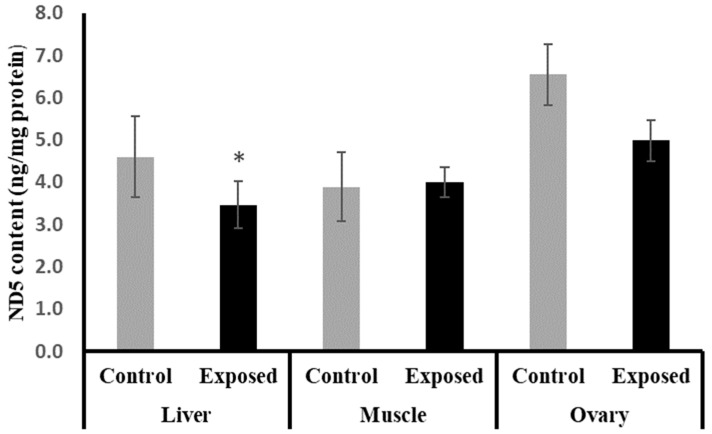
NADH dehydrogenase subunit-5 (ND5) contents in control and SOF-exposed female rats’ liver, muscle, and ovarian tissues. The data were indicated as Mean ± SD. 10 rats in each group. *: Statistically significant difference compared to the control group by Student *t*-test, *p* < 0.05.

The liver tissues of SOF-exposed female rats showed significantly lower ND5 content compared with normal rats by about −24.6 (*p* = 0.05). On the other hand, no significant differences were observed in the muscle and ovarian content of ND5 (*p* = 0.694, 0.084), respectively (Figure 6).

### 2.10. Mitochondrial DNA (mtDNA) Copy Number in Different Tissues

**Figure 7:**
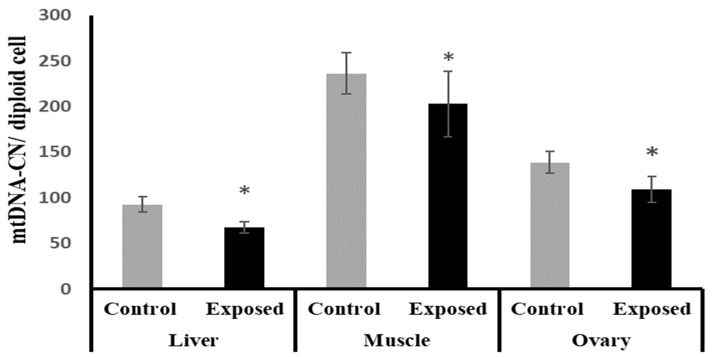
Mitochondrial DNA (mtDNA) copy number in control and SOF-exposed female rats’ liver, muscle, and ovarian tissues. The data were indicated as Mean ± SD. 10 rats in each group. *: Statistically significant difference compared to the control group by Student *t*-test, *p* < 0.05.

The liver, muscle, and ovarian tissues of SOF-exposed female rats showed significantly lower mtDNA copy numbers compared with normal female rats by about −27.1, −14.16, and −21.4% (*p* < 0.001, 0.022, *p* < 0.001), respectively (Figure 7).

### 2.11. Peroxisome Proliferator-Activated Receptor γ Coactivator-1 α (PGC-1α) Expression and Contents in Different Tissues

**Figure 8:**
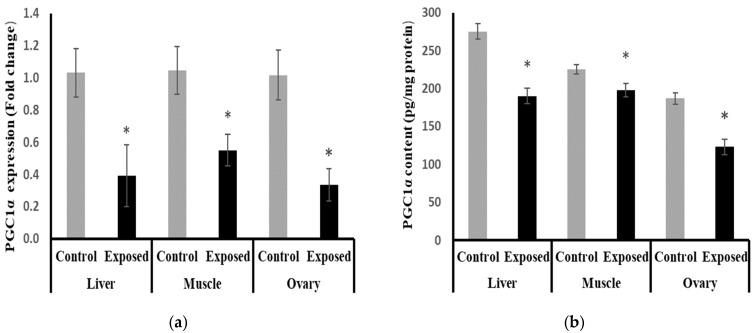
Peroxisome Proliferator-Activated Receptor γ Coactivator-1 α (PGC-1α) expression at mRNA (**a**) protein (**b**) levels in control and SOF-exposed female rats’ liver, muscle, and ovarian tissues. The fold change mRNA level vs. control. The data were indicated as Mean ± SD. Ten rats in each group. *: Statistically significant difference compared to the control group by Student *t*-test, *p* < 0.05.

At the mRNA level, the expression of PGC-1α was significantly reduced in the liver, muscle, and ovarian tissues from the SOF-exposed female rats by about −61.9, −47.5, and −67.09%, respectively, compared with the tissues from normal females (*p* < 0.001) (Figure 8a). At the protein level, liver, muscle, and ovarian tissues of SOF-exposed female rats showed a significant decrease in PGC-1a content compared with normal rats by about −30.9, −12.2, and −34%, respectively (*p* < 0.001) (Figure 8b).

### 2.12. Mitochondrial Transcription Factor-A (Tfam) Expression and Contents in Different Tissues

**Figure 9:**
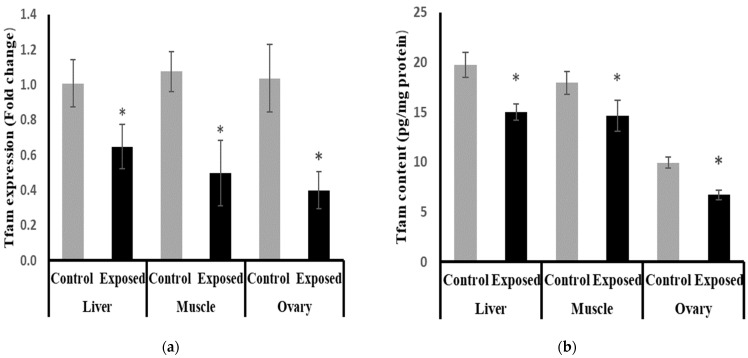
Mitochondrial transcription factor-A (Tfam) expression at mRNA (**a**) protein (**b**) levels in control and SOF-exposed female rats’ liver, muscle, and ovarian tissues. The fold change mRNA level vs. control. The data were indicated as Mean ± SD. Ten rats in each group. *: Statistically significant difference compared to the control group by Student *t*-test, *p* < 0.05.

The expression of Tfam was significantly suppressed in the liver, muscle, and ovarian tissues of SOF-exposed female rats by about −35.9, −53.9, and −61.49%, respectively, compared with tissues from normal female rats (*p* < 0.001) (Figure 9a).

The protein levels of Tfam were significantly lower in the liver, muscle, and ovarian tissues of SOF-exposed female rats compared with normal rats by about −24, −18.5, and −32.4%, respectively (*p* < 0.001) (Figure 9b).

### 2.13. Tissues Content of Nuclear Respiratory Factor 1 (NRF1)

**Figure 10:**
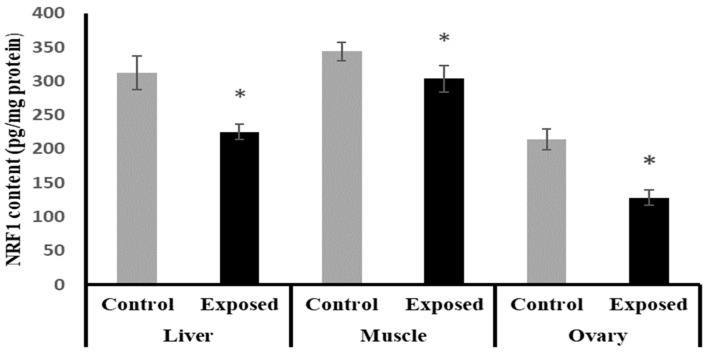
Nuclear respiratory factor 1 (NRF1) contents in control and SOF-exposed female rats’ liver, muscle, and ovarian tissues. The data were indicated as Mean ± SD. Ten rats in each group. *: Statistically significant difference compared to the control group by Student *t*-test, *p* < 0.05.

The NRF1 content in liver, muscle, and ovarian tissues of SOF-exposed female rats showed a significant decrease compared with normal rats by about −27.9, −11.6, and −40%, respectively (*p* < 0.001) (Figure 10).

### 2.14. Tissues Expression of DNA Polymerase γ (POLG)

**Figure 11:**
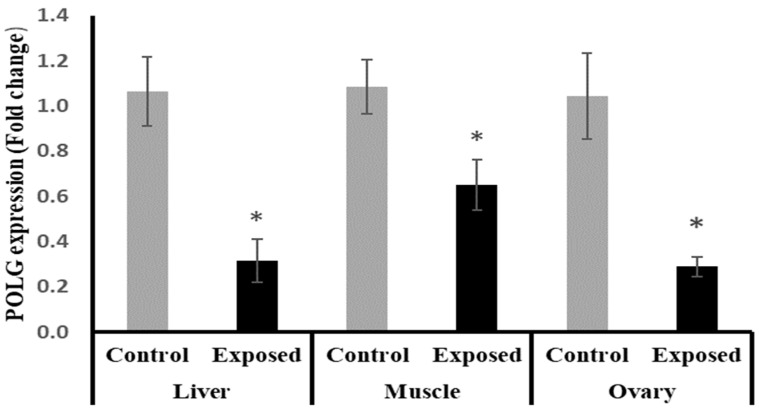
DNA polymerase γ (POLG) expression in control and SOF-exposed female rats’ liver, muscle, and ovarian tissues. The fold change mRNA level vs. control. The data were indicated as Mean ± SD. Ten rats in each group. *: Statistically significant difference compared to the control group by Student *t*-test, *p* < 0.05.

The results of POLG expression showed significant down-regulation in the liver and muscle of Sofosbuvir-exposed female rats by about −70.5 and −40%, respectively, compared with the control females. Also, the ovarian tissues showed significant down-regulation in POLG expression by about 72.3% compared with the normal females (*p* < 0.001) (Figure 11).

### 2.15. Nuclear Factor Kappa B (NF-κB) Content and Expression in Different Tissues

**Figure 12:**
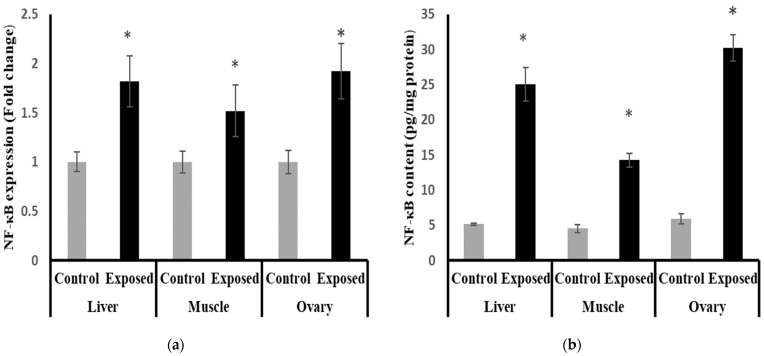
Nuclear Factor Kappa B (NF-κB) expression at mRNA (**a**) protein (**b**) levels in control and SOF-exposed female rats’ liver, muscle, and ovarian tissues. The fold change mRNA level vs. control. The data were indicated as Mean ± SD. Ten rats in each group. *: Statistically significant difference compared to the control group by Student *t*-test, *p* < 0.05.

The results of NF-κB expression showed significantly higher expression in liver, muscle, and ovarian tissues compared to normal rats, by about 71, 50, and 90%, respectively (*p* < 0.001) (Figure 12a).

The liver, muscle, and ovarian tissues of Sofosbuvir-exposed female rats showed a significantly high NF-κB content compared with normal rats by about 386.7, 214.7, and 408.8%, respectively (*p* < 0.001) (Figure 12b).

### 2.16. Correlation Studies

**Figure 13:**
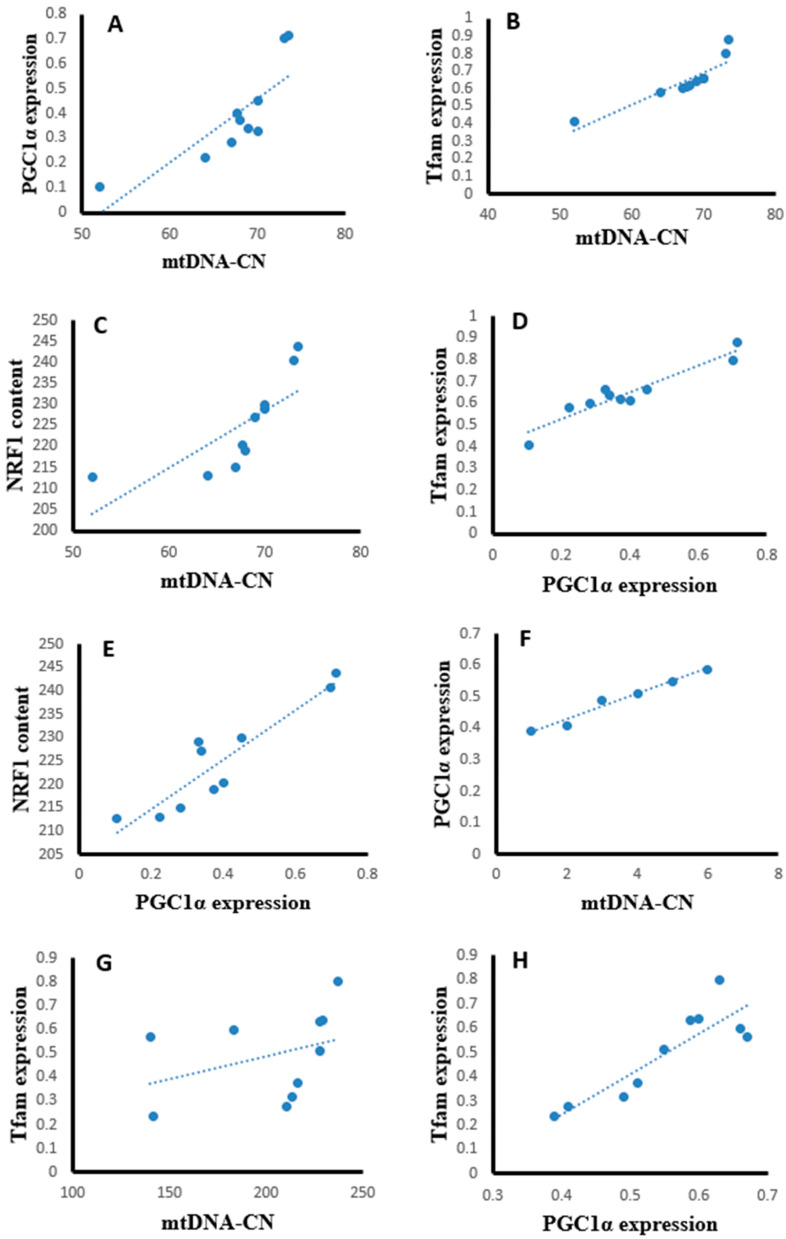

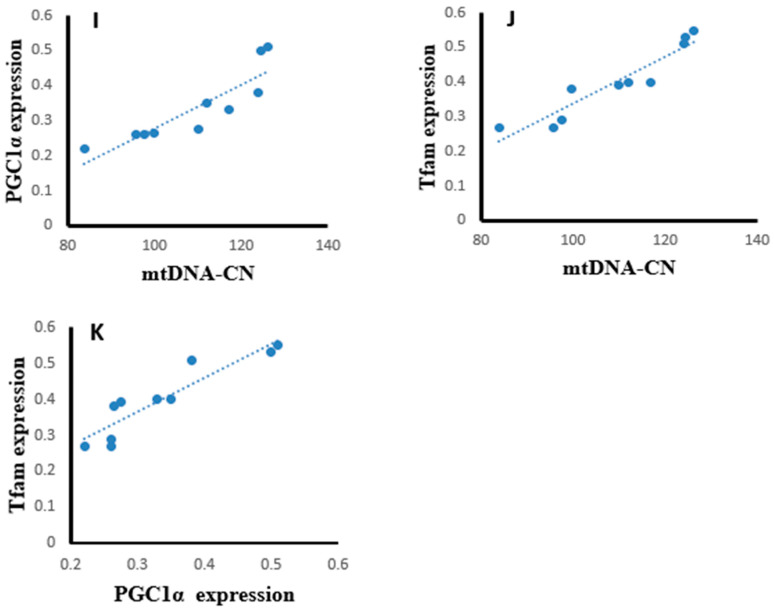
Correlation curves between different parameters in the different tissues of exposed female rats. (**A**–**E**) correlation curves in hepatic tissues between mtDNA copy number with (**A**) PGC-1α expression, (**B**) Tfam expression, and (**C**) NRF1 content; PGC-1α expression with (**D**) Tfam expression and (**E**) NRF1 content; (**F**–**H**) correlation curves in muscle tissues between mtDNA copy number with (**F**) PGC-1α expression and (**G**) Tfam expression; (**H**) PGC-1α expression with Tfam expression, (**I**–**K**) correlation curves in ovarian tissues between mtDNA copy number with (**I**) PGC-1α expression and (**J**) Tfam expression; and (**K**) PGC-1α expression with Tfam expression.

Statistical analysis using the Pearson correlation revealed that hepatic tissues of the SOF-exposed female rats showed positive significant correlation between mtDNA copy number with PGC-1α expression (r = 0.823, *p* = 0.003, Figure 13A), Tfam expression (r = 0.904, *p* = 0.001, Figure 13B), and NRF1 content (r = 0.763, *p* = 0.001, Figure 13C). The PGC-1α expression showed positive significant correlation with Tfam expression (r = 0.948, *p* = 0.001, Figure 13D) and NRF1 content (r = 0.922, *p* = 0.001, Figure 13E). In the muscle tissues of SOF-exposed female rats, the mtDNA copy number was positively correlated with PGC-1α expression (r = 0.952, *p* = 0.001, Figure 13F) and Tfam expression (r = 0.847, *p* = 0.002, Figure 13G). The PGC-1α expression showed positive significant correlation with Tfam expression (r = 0.979, *p* = 0.001, Figure 13H). In the ovarian tissues of SOF-exposed female rats, the mtDNA copy number was positively correlated with PGC-1α expression (r = 0.876, *p* = 0.001, Figure 13I) and Tfam expression (r = 0.937, *p* = 0.002, Figure 13J). the PGC-1α expression showed positive significant correlation with Tfam expression (r = 0.917, *p* = 0.001, Figure 13K).

## 3. Discussion

This present study raises major concerns regarding the safety of SOF treatment in young females. This study provides evidence of SOF-induced metabolic and mitochondrial changes in different tissues of female rats, including disturbed glucose and lipid homeostasis, modulated expression of the regulatory mechanisms controlling the mitochondrial biogenesis and functions, and depleted mtDNA copy number.

At the glucose homeostasis level, SOF induced a significant hypoglycemic effect in the exposed female rats, with no significant changes in the serum insulin level or the insulin resistance index (HOMA-IR). This effect was confirmed by a previous report, which indicated that the treatment of HCV-infected type 2 diabetic (T2DM) patients with the antiviral Sofosbuvir/ledipasvir results in symptomatic hypoglycemia despite the decrease in insulin dosage [11]. Due to this effect, it was recommended to reduce the dose of the anti-diabetic drugs in T2DM patients during treatment with direct-acting antiviral drugs. The cause of hypoglycemia could be due to HCV clearance and/or SOF metabolism in the liver.

The hypoglycemic effect of SOF was associated with a significant increase in the triglycerides and LDL-cholesterol levels compared with the control rats with no significant changes in the total and HDL-cholesterol. Similar effects were indicated in diabetic patients treated with SOF [11]. These findings imply that SOF shifts the serum lipid profile toward dyslipidemia and a subsequent risk of cardiovascular disease.

A significant elevation was found in the serum activities of ALT and AST in SOF-treated rats compared with control rats. The treatment of HCV patients with SOF significantly declined and normalized the serum ALT and AST activities, and this normalization of liver enzymes predicts the success of SOF treatment [12]. However, in our study, the animals were normal without infection and hepatitis. So, the increased serum liver enzymes may imply the hepatic damaging effect of SOF on the normal liver. On the other hand, SOF treatment has no significant effect on serum urea and creatinine.

Mitochondria are the powerhouse of the eukaryotic cell and play a central role in regulating cellular metabolism, proliferation, death, and signaling. The mitochondrial cell number is correlated with the mtDNA copy number, and mitochondrial biogenesis is under the control of a complex genetic system [13]. PGC-1α, Tfam, and NRF1 are the key transcription factors regulating a cascade of factors that contribute to the control of mitochondrial metabolism and biogenesis. In brief, PGC-1α binds to both NRF1 and NRF2. In turn, NRF1 interacts with certain promoter regions of the Tfam gene and increases its expression. Tfam plays a major role in mtDNA replication and transcription [14].

In the ovaries, SOF treatment caused a significant decline in the mtDNA copy number, which may imply impaired mitochondrial biogenesis. This impairment was associated with the marked suppression of the ovarian expression of PGC-1α and Tfam at mRNA and protein levels. Also, the protein level of NRF1 was downregulated. The ovarian tissues of the female rats exposed to SOF showed significant suppression of POLG, which is an essential element controlling mtDNA replication and repair. Also, the female rats treated with SOF showed a significant decline in ovarian citrate synthase activity and ND5 content. Citrate synthase (CS) is a rate-limiting enzyme in the citrate cycle that can catalyze the conversion of oxaloacetate and acetyl-CoA to citrate, and its activity is thought to be a biomarker of mitochondrial content and function [15]. Subunit 5 of NADH dehydrogenase (ubiquinone, known as Complex I) is located in the mitochondrial inner membrane and is encoded by MT-ND5 (a gene of the mitochondrial genome), which is considered a hotspot for disease-causing mutations [16]. Complex I is the largest of the respiratory chain complexes in the inner mitochondrial membrane and plays an essential role in the electrons’ flow in the ETC and coupled ATP synthesis [17]. So, the decline in ND5 content and CS activity may result in impaired ETC and ATP synthesis in the ovarian tissues. Since mitochondria are essential for oocyte quality, defective mitochondrial functions and/or biogenesis in the ovarian tissue can affect the quality of oocytes, which may result in serious adverse effects on embryo development and pregnancy outcome [18]. The ovarian mitochondria play an important role in the fertilization and maturation of the oocytes, as well as embryonic development because it is involved in the assembly of the meiotic spindle, chromosomal segregation, fertilization, cell division, and embryo development [19]. The low ovarian mtDNA copy number and lower ND5 content may be considered markers of poor oocyte quality, which may lead to poor embryonic development [18]. Moreover, these side effects may be transmitted to subsequent generations and cause many diseases [20].

The mechanism of Sofosbuvir-induced alterations in mitochondrial biogenesis and functions is unclear and needs further investigation. However, the direct or indirect action of the nuclear regulators of mitochondrial biogenesis, including PGC-1α/NRF1/Tfam, on the redox status of the tissues and the inflammatory pathways cannot be excluded. Our results indicated a Sofosbuvir-induced suppression of PGC-1α/NRF1/Tfam in the ovarian tissues at both mRNA and protein levels; an induction of a state of oxidative stress, as indicated by the elevation in the lipid peroxidation index (MDA) and DNA oxidation biomarker (8-OHdG); impairment of the antioxidant system, as indicated by the suppressed expression of Nfe2l2 (nuclear factor erythroid 2-related factor 2, the master transcription factor that regulates the expression of endogenous antioxidants); and an induction of inflammation, as indicated by induced NF-κB expression. All of these effects may increase the frequency of mtDNA mutations, leading to mitochondrial dysfunction [21]. Also, SOF may shift the hormonal homeostasis of the female rats, which may affect the ovarian metabolism. This present study showed that the female rats exposed to SOF have significantly lower serum levels of FSH by about 30% compared with control rats, while the level of LH non-significantly increased by about 12%. Shen et al. (2016) reported that FSH plays an important role in maintaining mitochondrial integrity and relieves oxidative stress-induced cell death in mice ovarian granulosa cells [22].

The liver tissue of rats exposed to SOF showed a similar pattern of changes as the ovarian tissue. The female rats exposed to SOF showed significantly lower tissue citrate synthase activity and ND5 content; suppressed PGC-1α, Tfam mRNA, and protein levels; and decreased POLG expression compared with the control rats. Also, the protein level of NRF1 is downregulated. In line with our data, the treatment of different cell lines with SOF results in the downregulation of the genes that interfere with mitochondrial function [23]. Also, the mtDNA copy number was significantly decreased in the liver tissue. The liver tissues of Sofosbuvir-exposed female rats showed induced NF-κB expression, high MDA and 8-OHdG contents, and suppressed Nfe2l2 expression, which might indicate a state of inflammation and oxidative stress that could contribute to the development and progression of liver diseases.

In the liver, mitochondria are the main energy conversion sites [24]. So, any external factor or drugs that affect the mitochondrial dynamics may have a serious impact on the overall metabolic homeostasis and may predispose to serious diseases such as cancer and metabolic diseases. In this present study, the Sofosbuvir-induced depletion in hepatic mtDNA copy number may result in the reduction of ND5 concentrations, leading to impaired mitochondrial ETC activity and causing an accumulation of electrons in the early stages of the ETC cellular, leading to excessive ROS production and oxidative stress [25,26]. Oxidative stress can damage ETC proteins, resulting in a shutdown of energy production, i.e., inhibited ETC. The inhibited ETC may impair the mitochondrial β-oxidation with the subsequent accumulation of fatty acids and lipids in the liver, causing steatohepatitis, which may shift the serum lipid profile toward dyslipidemia and cardiovascular risk. As it is near to the ETC, mtDNA is susceptible to oxidative damage, which may alter mitochondrial gene expression and mutations that are implicated in many diseases [27]. Low hepatic CS activity is associated with impaired glucose tolerance and abnormalities in lipid metabolism as it leads to the accumulation of subcutaneous fat [15].

PGC-1α is highly expressed in tissues with high energy demands, such as liver and muscle tissues. The impaired expression and functions of PGC-1α are associated with the pathogenesis of metabolic syndrome, including obesity, T2DM, cardiovascular disease, and hepatic steatosis [28]. PGC-1α regulates the expression of antioxidant genes, including manganese superoxide dismutase, catalase, peroxiredoxin, uncoupling protein 2, thioredoxin 2, and thioredoxin reductase, and, thus, it acts as a protective pathway to prevent oxidative injury and mitochondrial dysfunction. The impaired expression of hepatic PGC-1α alters redox homeostasis and induces an inflammatory response and metabolic disturbances [29].

Because PGC-1 α coordinates mitochondrial biogenesis and function, the suppressed PGC-1a expression in the liver is expected to lead to impaired mitochondrial function and biogenesis, leading to decreased fatty acid oxidation and the accumulation of triglyceride development of non-alcoholic fatty liver diseases (NAFLDs). In line with the previous suggestion, the study by Balampanis et al. (2019) showed that the hepatic expression of PGC-1α is negatively correlated with fat content in obese patients [30]. Also, an inverse correlation was found between hepatic PGC-1α expression and the hepatic fat and disease severity in animal models and patients with NAFLD [31]. Moreover, the hepatic PGC-1α expression is negatively associated with serum AST and ALT. This finding implies a beneficial role for PGC-1α as a protective factor against hepatic steatosis both in vivo and in vitro [32,33].

The results of this present study indicated that muscle tissues are also affected by Sofosbuvir-induced mitochondrial changes, as we detected the suppressive effects of SOF exposure on the mtDNA copy number, NRF1, PGC-1α, Tfam, and POLG. Jin et al. (2017) demonstrated that the structurally diverse ribonucleoside analogs favipiravir, balapiravir, Sofosbuvir, and others cause mitochondrial toxicity in the cells [10]. Also, SOF exposure causes inflammation in skeletal muscles, as shown by induced NF-κB expression and increased oxidative stress demonstrated by an elevation in MDA and 8-OHdG contents and a decline in the Nfe2l2 expression.

Skeletal muscle plasticity is important for adaptation to various physiological conditions. Physiological stimuli include alterations in physical activity and workload. The response of skeletal muscle is via alterations in contractile machinery, calcium management, and energy capacity. To match skeletal muscle demands, mitochondrial ATP production is tightly regulated. Consequently, the skeletal muscle mitochondrial coordination has developed for this function. Suppressions in mitochondrial biogenesis and ATP production are implicated in many diseases [34]. Mitochondrial respiratory capacity, as judged by CS activity, may be impaired with age. Some investigators have found CS activity to decline with age in skeletal muscles, which is significantly correlated with higher levels of mitochondrial oxidative damage [35].

The possible mechanisms of SOF in impairing a broad number of enzymes participating in energy production pathways were observed in our recent study [36]. The increased DNA oxidative damage may be mutagenic, especially in the mitochondria, due to the close vicinity of mtDNA to the source of ROS. ND5 may be a target of this mutagenicity that results in unfunctional ND5, which may impair the ETC and further increase ROS production. Also, CS downregulation induces a reduction in ATP production, as well as an increase in oxidative stress and cell apoptosis. Moreover, the induction of mitochondrial dysfunction increases oxidative stress with reduced CS activity, ATP production, and mitochondrial biogenesis. The crosstalk between different transcription factors, i.e., PGC-1α, Tfam, and NRF1, in our study was confirmed by the correlation findings. The PGC-1α showed a positive significant correlation with Tfam expression and NRF1 in the liver and muscle tissues of SOF-exposed female rats. Also, the interplay between mtDNA copy numbers and those transcription factors was supported by the correlation data, as mtDNA copy number showed a positive significant correlation with PGC-1α expression, Tfam expression, and NRF1 content.

## 4. Materials and Methods

### 4.1. Experimental Animals

Twenty female Wistar albino rats weighing 115–125 g aged about 2 months old were used in this study. Rats were purchased from the Medical Research Institute’s animal house, Alexandria University, Egypt. Five animals were housed in a cage at a temperature of 23 °C with a 12 h light/12 h dark cycle, standard humidity, and access to food and drink.

### 4.2. Ethical Statement

This study was approved by the Alexandria University Institutional Animal Care and Use Committee (AlexU-IACUC, Approval number: AU01219073023). All experiments fulfill the guidelines of the National Institutes of Health guides for the care and use of laboratory animals (NIH Publications No. 8023, revised 1978) and the recommendations of Egypt’s guide for the care and use of laboratory animals. All efforts were made to curb the distress of rats during the experimental period. Using best practices for commonly used procedures, such as blood sampling and feeding rats with a consistent supply of food and water, and simultaneously cleaning their cages, can enormously improve animal welfare.

### 4.3. Drug

Sofosbuvir (SOF) (a drug produced by Pharco Pharmaceuticals in Alexandria, Egypt), was available in the form of tablets with the commercial name ‘Gratisovir’. Each tablet containing 400 mg of SOF was dissolved in distilled water and administrated orally to the rats via a gastric tube at a dose of 4 mg/kg/day for 3 months [37,38].

### 4.4. Experimental Design

All rats were fed standard rat chow during the experiment (3.1 kcal/g, 58.0% CHO, 18.0% fat, 24.0% protein) and were randomly divided into two groups: the control group—10 healthy female rats that were maintained under a normal diet and received placebo (distilled water) for three months, and the exposed group—10 healthy female rats that were supplemented with 4 mg/kg/day of SOF drug orally by gastric tube for three months [37,38]. The health and behavior of the animals were observed daily by visual inspection, and any rats that died were excluded from the experiment periods. The death rate (10%) was the same in the two groups. Only one rat died from each group without any illness from the drug.

### 4.5. Collection of Samples

At the end of the experiment, all animals were exposed to overnight fasting and then sacrificed under deep anesthesia with ketamine 100 mg/kg and xylazine 10 mg/kg. Blood samples were subsequently collected and centrifuged at 3000× *g* for 10 min to determine serum glucose, insulin, lipid profile, urea, and creatinine levels. The liver, muscle, and ovarian tissues were collected and washed, and each organ was divided into three aliquots: the first one was used for DNA isolation for the assessment of mtDNA copy number and 8-OhdG, the second was used for total RNA isolation for the assessment of gene expression, and the third aliquot was homogenized in phosphate-buffered saline (0.1 M, pH 7.4) in ratio 1:9 and centrifuged at 10,000× *g* for 10 min at 4 °C. The supernatant was stored in aliquots for subsequent determinations of total protein level by Lowry’s method, and MDA, citrate synthase activity, and the protein levels of ND5, PGC-1α, Tfam, NRF1, and NF-κB by ELISA.

### 4.6. Serum Parameters

Serum levels of FBG, urea, creatinine, ALT, AST, TG, TC, and HDL-C were analyzed using commercially accessible kits (Bio-Med Diagnostic INC, White City, OR, USA). LDL-C was estimated according to Friedewald’s equation, LDL-C (mg/dL) = TC − (HDL-C) − (TG/5).

An immunoassay kit (EMD Millipore, Burlington, MA, USA) was used to measure LH, FSH, and insulin serum levels of LH, FSH, and insulin. The HOMA-IR was calculated afterward using the formula shown below [39]:

HOMA-IR = (Fasting insulin((µIU)/mL) × Fasting glucose(mg/dL))/(22.5 × 18)

### 4.7. Tissues Citrate Synthase Activity

Citrate synthase activity can be determined through the production of the -SH group released from CoA-SH using the reactive Ellman reagent (5,5′-dithiobis [2-nitrobenzoic] DTNB) and monitoring the absorbance at 412 nm. Citrate synthase activity can be calculated according to the following formula [40]:

Citrate synthase activity (U/mg protein) = ΔA/minute/(ε × L × mg protein in the assay)

where ε is the extinction coefficient of TNB at 412 nm = 13.6 mM^−1^cm^−1^, L is the path length for absorbance (cm).

### 4.8. ELISA Parameters

Specific rat ELISA kits were used for the assessment of ND5 (Chongqing Biospes Co., Chongqing, China), PGC-1α, Tfam, NRF1 (MyBioSource, San Diego, CA, USA), and NF-κB (CUSABIO Co., Houston, TX, USA) following the manufacturer’s recommendations. Using Lowry’s method, the concentration of total protein was determined.

### 4.9. Tissues Mitochondrial DNA (mtDNA) Copy Number

To determine the abundance of mtDNA in relation to nuclear DNA, we used a qPCR assay. Upon complete genomic DNA isolation, we utilized a specific primer pair for mtDNA sequence (mtDNA) and a primer pair specific for nuclear sequence (PGC-1α) to perform the same number of PCR cycles and calculate the relative mtDNA signal to nuclear DNA signal. The nuclear gene was utilized to measure nuclear DNA (nDNA), and therefore, normalization of the mtDNA amount per the nDNA of the diploid cells using the following equation:

R = 2^−ΔCt^ where ΔCt = Ct _mtDNA_ − Ct _nuclear_

The total DNA was extracted from the various tissues using a Dneasy kit (Qiagen, Hilden, Germany) according to the manufacturer’s instructions. A specific primer pair for mtDNA (forward: 5′-ACACCAAAAGGACGAACCTG-3′ and reverse: 5′-ATGGGGAAGAAGCCCTAGAA-3′) and a primer pair for the nuclear PGC-1α gene (forward: 5′-ATGAATGCAGCGGTCTTAGC-3′ and reverse: 5′-AACAATGGCAGGGTTTGTTC-3′) were used. PCR reactions were carried out using SYBR Green PCR Master Mix (Qiagen, Hilden, Germany), 0.5 μM of each primer pair, and 50 ng genomic DNA under the following conditions: 95 °C for 10 min followed by 40 cycles of 95 °C for 15 s, 60 °C for 30 s, and 72 °C for 30 s [41].

### 4.10. Tissue mRNA Expression of PGC-1α, Tfam, Nfe2l2, NF-κB, and POLG

A total of 30 mg of liver, muscle, or ovarian tissues were used for total RNA isolation by using the Rneasy kit (Qiagen, Hilden, Germany) following the manufacturer’s recommendations, and the amount and quality of extracted RNA were confirmed using nanodrop. The extracted RNA was reverse transcripted using the Maxime™ RT PreMix kit (iNtRON Biotechnology Inc., Seongnam-si, Republic of Korea) following the manufacturer’s recommendations. The tissue expression of PGC-1α, Tfam, Nfe2l2, NF-κB, and POLG were quantified in the cDNA by Rotor-Gene Q qPCR (Qiagen, Hilden, Germany) using QuantiTect SYBR Green PCR Master Mix (Qiagen, Hilden, Germany). Quantitative PCR amplification conditions were adjusted as an initial denaturation at 95 °C for 10 min, and then 45 cycles of PCR for amplification as follows: denaturation at 95 °C for 20 s, annealing at 55 °C for 20 s, and extension at 70 °C for 15 s. The housekeeping gene glyceraldehyde 3-phosphate dehydrogenase (GAPDH) was used as a reference gene for normalization. The primers used for the determination of rat genes are presented in Table 4. The relative change in mRNA expression in samples was estimated using the 2^−ΔΔCt^ method.

### 4.11. Determination of Malondialdehyde (MDA) as Thiobarbituric Acid Reactive Substances (TBARS)

The Draper and Hadley method was used to measure malondialdehyde. The tissue samples were heated with thiobarbituric acid (TBA) at low pH. The pink chromogen that results had a maximum absorbance of 532 nm [42].

### 4.12. Determination of 8-Hydroxy Deoxyguanosine (8-OHdG)

The isolated DNA samples from the liver, muscle, and ovarian tissues were enzymatically digested, as described previously [38,43], and used for the assessment of the 8-OHdG content using an ELISA kit (Chongqing Biospes Co., Ltd. Chongqing, China) following the manufacturer’s recommendation.

### 4.13. Statistical Analysis

The SPSS software package, version 18.0 (SPSS, Chicago, IL, USA), was used to analyze the data. The data were described as Mean ± SD and analyzed using the Student independent *t*-test (two-tailed) to compare between different groups. The *p*-value was assumed to be significant at *p* < 0.05. Using the Pearson correlation coefficient, the correlation coefficients (r) between various tested parameters were examined; *p* < 0.05 was considered the significance limit for all comparisons.

## 5. Conclusions

This present study represents a pilot finding that points out the Sofosbuvir-induced adverse effects on different tissues of young female rats. These effects are mediated through the suppression of the regulatory machinery of mitochondrial biogenesis and mitochondrial functions, impairment of antioxidants, and induction of oxidative stress and inflammation. Although these results are potentially interesting, we recommend further studies to confirm these results by testing the altered mitochondrial transcriptional factors, the increased oxidative stress, and the increased inflammation by immunohistochemistry and mitochondrial electron microscopic examination.

## Figures and Tables

**Table 1:** The change of glucose homeostasis parameters in control and SOF-exposed female rats.

Groups	Fasting Blood Glucose (mg/dL)	Insulin (µIU/mL)	HOMA-IR
Control	60.00 ± 5.58	5.886 ± 0.86	0.87 ± 0.17
SOF-exposed	44.46 * ± 6.11	6.74 ± 1.3	0.74 ± 0.17
% of change from control	−25.6	13.26	−16.4

The data were indicated as Mean ± SD. Ten rats in each group. *: Statistically significant difference compared to control group by Student *t*-test, *p* < 0.05 (HOMA-IR: Homeostasis model of assessment insulin resistance index).

**Table 2:** The change of lipid profile parameters in control and SOF-exposed female rats.

Groups	TG (mg/dL)	TC (mg/dL)	HDL-C (mg/dL)	LDL-C (mg/dL)
Control	111.5 ± 6.52	81.2 ± 13.05	44.3 ± 6.91	14.6 ± 8.97
SOF-exposed	144.5 * ± 16.87	162.5 * ± 23.50	39.4 ± 10.11	94.2 * ± 24.4
% of change from control	29.6	100	−11	545.2

The data were indicated as Mean ± SD. 10 rats in each group. *: Statistically significant difference compared to the control group by Student *t*-test, *p* < 0.05. TG: Triglyceride, TC: Total Cholesterol, HDL-C: High-density lipoprotein-cholesterol, LDL-C: Low-density lipoprotein-cholesterol.

**Table 3:** The change in serum alanine aminotransferase (ALT) and aspartate aminotransferase (AST) activities and serum urea and creatinine levels in control and SOF-exposed female rats.

Groups	ALT (U/L)	AST (U/L)	Urea (mg/dL)	Creatinine (mg/dL)
Control	32.2 ± 4.37	62.9 ± 15.22	47.80 ± 4.78	0.8 ± 0.19
SOF-exposed	50.3 * ± 4.57	83.60 * ± 14.77	53.70 * ± 5.62	0.98 ± 0.22
% of change from control	56.2	32.9	12.3	22.6

The data were indicated as Mean ± SD. 10 rats in each group. *: Statistically significant difference compared to the control group by Student *t*-test, *p* < 0.05.

**Table 4:** Primers used for qRT-PCR.

Gene	Accession Number	Primer Sequence	
*PGC-1α*	NM_031347.1	F:	5′-GTGCAGCCAAGACTCTGTATGG-3′
		R:	5′-GTCCAGGTCATTCACATCAAGTTC-3′
*POLG*	NM_053528.1	F:	5′-GGACCTCCCTTAGAGAGGGA-3′
		R:	5′-AGCATGCCAGCCAGAGTCACT-3′
*Tfam*	NM_031326.2	F:	5′-CCCACAGAGAACAGAAACAG-3′
		R:	5′-CCCTGGAAGCTTTCAGATACG-3
*NF-κB (P65)*	NM_199267.2	F:	5′-CAGGACCAGGAACAGTTCGAA-3′
		R:	5′-CCAGGTTCTGGAAGCTATGGAT-3′
*Nfe2l2*	NM_031789.2	F:	5′-CAAATCCCACCTTGAACACA-3′
		R:	5′-CGACTGACTAATGGCAGCAG-3′
*GAPDH*	NM_017008.4	F:	5′-AATTGCAGCCATGTGGAGG-3′
		R:	5′-AGTTGTCATGGATGACCTTGG-3′

F: Forward, R: Reverse. PGC-1α: Peroxisome proliferator-activated receptor gamma coactivator-1 alpha, POLG: DNA polymerase gamma, TFAM: mitochondrial transcription factor A, NF-κB (P65): Nuclear Factor Kappa B subunit P65, Nfe2l2: nuclear factor erythroid 2-related factor 2, GAPDH: GAPDH glyceraldehyde-3-phosphate dehydrogenase.

## Data Availability

The data are available upon request.
